# Supplementary figures and images for: Striatal-Inoculation of α-Synuclein Preformed Fibrils Aggravated the Phenotypes of REM Sleep without Atonia in A53T BAC-SNCA Transgenic Mice
Source: Int J Mol Sci. 2022 Nov 2;23(21):13390. doi: 10.3390/ijms232113390 (PMC9656146; doi:10.3390/ijms232113390)

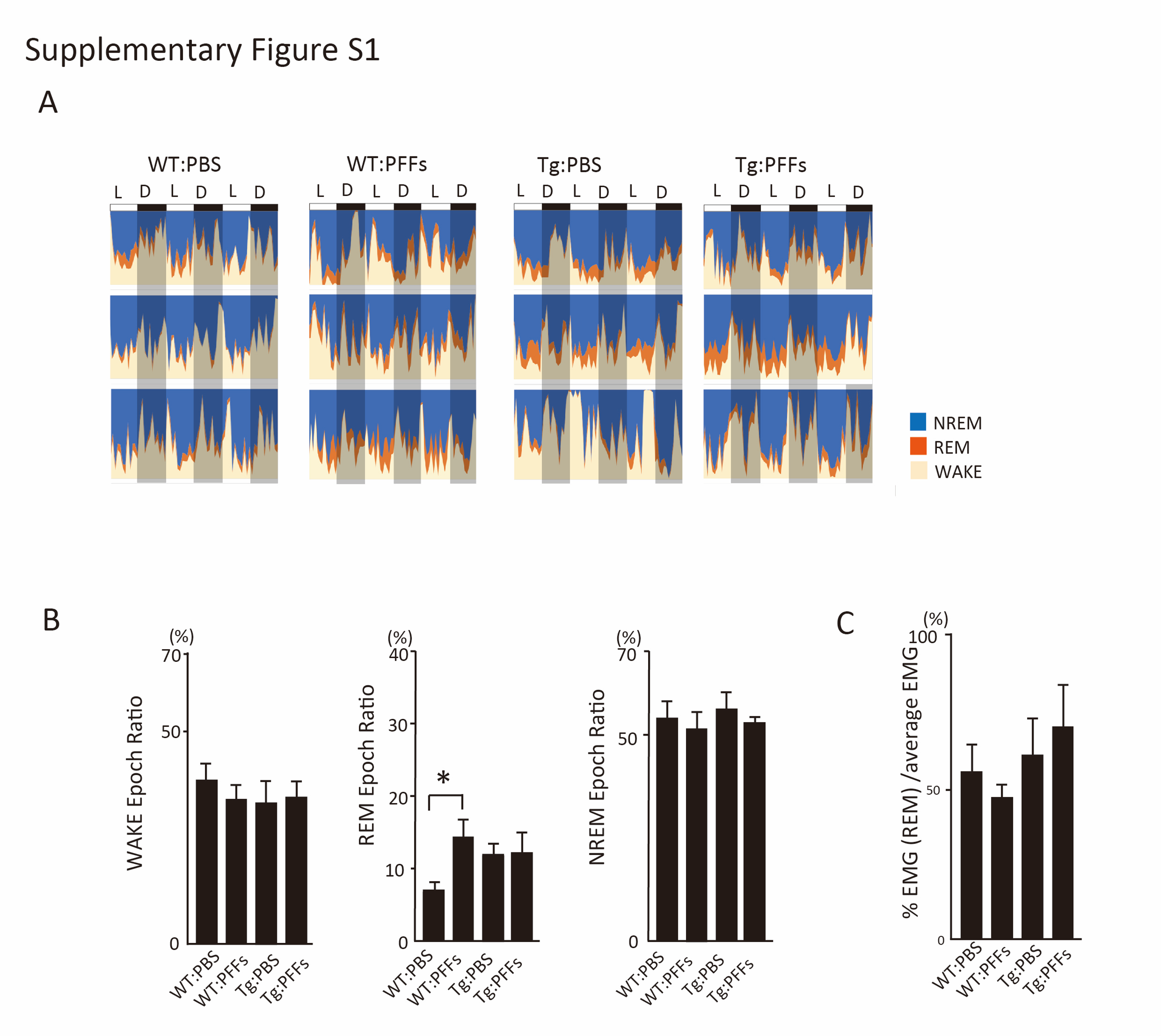

Supplement: Supplementary file 1 [file ijms-23-13390-s001.zip › Supplementary Figure S1.png]
